# Selection footprints reflect genomic changes associated with breeding efforts in 56 cucumber inbred lines

**DOI:** 10.1038/s41438-019-0209-4

**Published:** 2019-11-15

**Authors:** Bin Liu, Dailu Guan, Xuling Zhai, Sen Yang, Shudan Xue, Shuying Chen, Jing Huang, Huazhong Ren, Xingwang Liu

**Affiliations:** 1 Beijing Key Laboratory of Growth and Developmental Regulation for Protected Vegetable Crops, College of Horticulture, China Agricultural University, Beijing, 100193 P. R. China; 2 Centre for Research in Agricultural Genomics (CRAG), CSIC-IRTA-UAB-UB, Campus Universitat Autònoma de Barcelona, Bellaterra, 08193 Spain; 3 Department of Agronomy, College of Agriculture, Purdue University, West Lafayette, IN 47907 USA

**Keywords:** Agricultural genetics, Plant breeding

## Abstract

Cucumber selective breeding over recent decades has dramatically increased productivity and quality, but the genomic characterizations and changes associated with this breeding history remain unclear. Here, we analyzed the genome resequencing data of 56 artificially selected cucumber inbred lines that exhibit various phenotypes to detect trait-associated sequence variations that reflect breeding improvement. We found that the 56 cucumber lines could be assigned to group 1 and group 2, and the two groups formed a distinctive genetic structure due to the breeding history involving hybridization and selection. Differentially selected regions were identified between group 1 and group 2, with implications for genomic-selection breeding signatures. These regions included known quantitative trait loci or genes that were reported to be associated with agronomic traits. Our results advance knowledge of cucumber genomics, and the 56 selected inbred lines could be good germplasm resources for breeding.

## Introduction

Cucumber (*Cucumis sativus* L.; 2*n* = 2*x* = 14) is generally considered to be an economically important vegetable crop worldwide^1^, as well as a well-characterized model for studying fleshy fruit development^2^. Cultivated cucumber varieties have evolved from their wild progenitors under natural and artificial selection^3,4^. The domestication of cucumber began ~3000 years ago, and since then, different breeding strategies have been used to produce desired characteristics in order to meet the demand for human nutrition and health^5^. However, it was reported that cucumber had a low species diversity due to narrow bottlenecks^3^; therefore, the breeding of new cucumber varieties that require low inputs and are environmentally sustainable will probably still be challenging in the future.

Traditionally, the breeding of cucumber germplasm was accomplished simply by selecting lines with desirable characteristics for propagation, while the new methodology has accompanied the development of high-volume parallel genotyping and sequencing technologies, for instance, genomic selection^6^. However, selective cucumber breeding employing genomic markers was initiated only in the 1980s^5,7–9^, and the implementation of genomic selection in cucumber breeding is still difficult. Although artificial selection has greatly increased cucumber productivity and quality with expected improvements in traits, such as gynoecy, disease resistance, uniform ripening and bitterness^10–13^, genetic gains in genomic selection are pending. Next-generation DNA sequencing technologies now allow cost-effective genome sequencing at a population scale, which has led to the construction of variation maps for crop plants such as maize^14^, rice^15^, soybean^16^, sorghum^17^, apple^18^, watermelon^19^, pepper^20^, and cucumber^3^. Meanwhile, genome-wide association studies (GWASs) are used to detect genetic variations that underlie many important and complex traits in plants^21^. To date, some loci or genes have been identified by genomic studies in cucumber with large natural populations^3,22,23^. However, very few studies have identified loci or genomic regions in populations exposed to long-term artificial selection.

In recent decades, we focused on breeding elite cucumbers with outstanding phenotypes by crossing excellent Asian and European germplasm and performing artificial selection. On the one hand, we aimed to combine desirable agronomic traits from Asian and Eurasian lines in the progenies. On the other hand, we always selected the seeds from healthy plants and removed the disease-infected plants; therefore, most of the lines performed well in the field and were free of disease infection. As a result of long-term selection, we obtained 56 elite cucumber lines that retained phenotypic diversity. In this case, we would like to know the genomic characterizations and changes associated with this breeding history and the potential of our breeding materials.

Here, we resequenced the 56 cucumber lines and found that their genetic background was significantly different from that previously reported for 115 cucumber lines^3^. The data from our 56 lines revealed specific selected regions related to breeding efforts, and these selected regions were associated with the agronomic performance of cucumber varieties and harbored many reportedly important genes.

## Results and Discussion

### Determination of cucumber genetic structure with artificially selected inbred lines

**Fig. 1:**
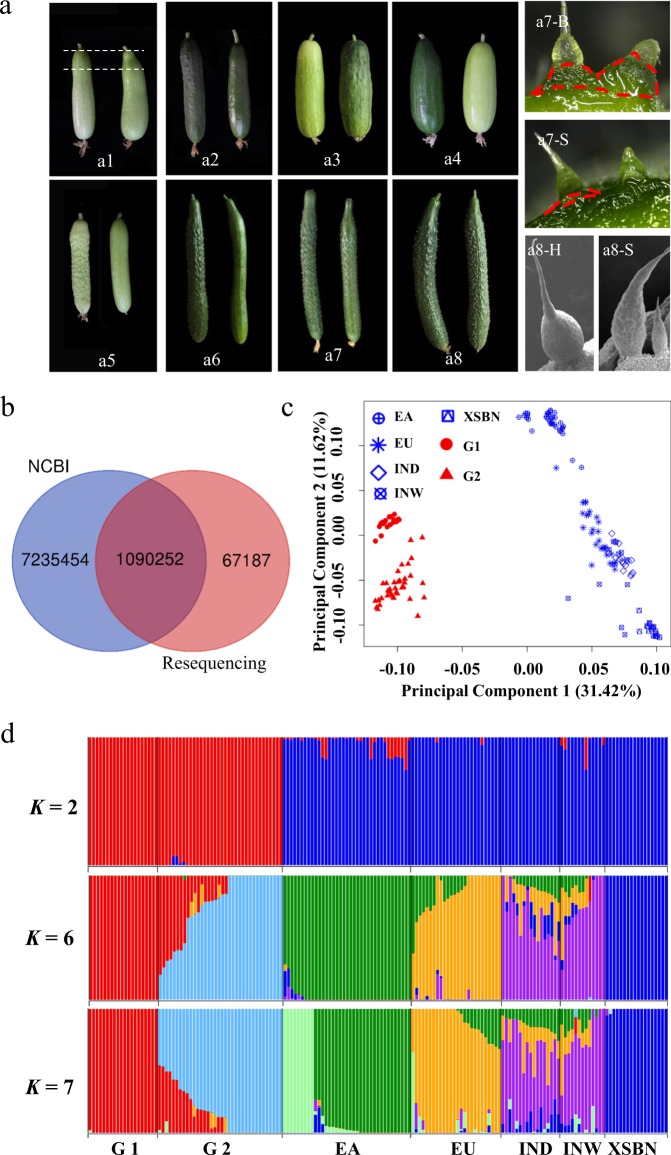
Determination of cucumber genetic structure and genomic variations in 56 artificial selection inbred lines. **a** Representatives of 56 cucumber lines with different phenotypes: (a1) Normal vs abnormal fruit shoulder (the part between the two white dotted lines). (a2) Dull vs glossy skin. (a3) Uniform vs nonuniform fruit color. (a4) Green vs yellow-green skin. (a5) Trichome and tubercule presence vs trichome and tubercule absence in yellow-green-skinned fruit. (a6) Trichome and tubercule presence vs trichome and tubercule absence in green-skinned fruit. (a7) Large tubercules (a7-B) vs small tubercules (a7-S). The tubercules are marked by red dotted lines. (a8) Hard spines (a8-H) vs soft spines (a8-S). **b** Venn diagram depicting unique and shared SNPs between the 56 resequenced lines and 115 previously reported lines. **c** Principal component analysis (PCA) of the 171 cucumber lines. The PCA considered principal components 1 (PC1) and 2 (PC2), which explained 31.42% and 11.62% of the variance, respectively. **d** ADMIXTURE analysis (*K* = 2, 6, and 7). Each bar represents one individual, and the length of the colored bar represents the proportion of the cucumber genome inherited from each ancestral population. East Asian (EA); Eurasian (EU); Indian domesticated (IND); Indian wild (INW); Xishuangbanna (XSBN); group 1 (G1); group 2 (G2)

The genetic data of 56 lines subjected to artificial selection represent both genomic variations and their usefulness in cucumber breeding improvement. According to our breeding aims, the 56 lines were assigned to group 1 (G1), which has a background similar to that of East Asian (EA) cucumber lines, and group 2 (G2), which has the Eurasian (EU) background (Supplementary Table 1; Supplementary Fig. 1). These lines were selected to obtain fruit traits with commercial value, such as normal vs abnormal fruit shoulder (Fig. 1 a1), dull vs glossy fruit skin^24^ (Fig. 1 a2), uniform vs nonuniform fruit color^12^ (Fig. 1 a3), green vs yellow-green fruit color (Fig. 1 a4), trichome and tubercule presence vs trichome and tubercule absence^25,26^ (Fig. 1 a5–6), large vs small tubercules^27^ (Fig. 1 a7, a7-B a7-S), and hard vs soft spines^28^ (Fig. 1 a8, a7-H a8-S). To generate the 56 lines, at least 13 founder lines from the EA or EU market were selected for cross-fertilization. Next, backcrossing or selfing was performed to obtain recombinant inbred lines or near-isogenic lines with the target traits.

We also included 115 lines sequenced by Qi et al. that were publicly available from the National Center for Biotechnology Information (NCBI, https://www.ncbi.nlm.nih.gov/)^3^. Resequencing of the 56 selected lines generated a total of 1.6 billion paired-end reads, with an average depth of ~18× and coverage of 98.4% (Supplementary Table 1). By aligning paired-end reads against the reference genome of the inbred cucumber line 9930^1^, a total of ~8.4 million single nucleotide polymorphism (SNPs) were identified (Fig. 1b). Among these SNPs, 67187 were unique in our breeding lines, indicating that the artificial selection lines were enriched with cucumber genomic variations, which are likely important for cucumber breeding.

To examine the genetic structure of the 56 inbred lines, principal component analysis (PCA)^29^ and ADMIXTURE analysis^30^ were employed. It was clear that our cucumber lines formed groups different from the previously published EA, EU, Indian domesticated (IND), Indian wild (INW) and Xishuangbanna (XSBN) groups (Fig. 1c, d, Supplementary Figs. 2 and 3). Along the first component, explaining 31% of the variation, our 56 selected lines clustered away from the others (Fig. 1c), which was supported by ADMIXTURE analysis when *K* = 2 (Fig. 1d). Within the 56 lines, separation was found between the two groups along the second component in the PCA and at *K* = 6 in the ADMIXTURE analysis (Fig. 1c, d). When the *K*-value with the lowest cross-validation error (*K* = 7) was used, the G1, G2, EA, EU, IND, INW, and XSBN groups were assigned to independent clusters (Fig. 1d). However, the EA cluster was divided into two different genetic backgrounds, which was somewhat different from the pattern observed with published data^3^ (Fig. 1d). In addition, this kind of clustering was also reinforced in the constructed neighbor-joining (NJ) tree^31^ (Supplementary Fig. 2), indicating that our 56 cucumber lines had a different genetic structure than the 115 lines^3^ obtained by selective breeding and suggesting that G1 and G2 represent ideal populations for cucumber breeding.

### Evidence of introgression and genetic diversity in two groups of cucumber

**Fig. 2:**
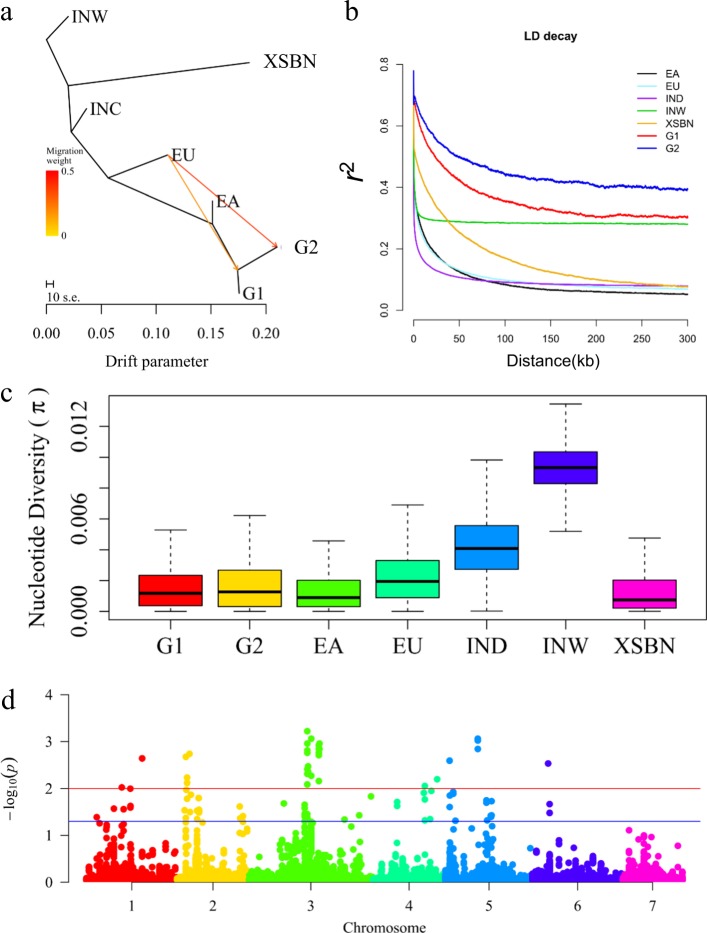
Evidence of introgression and genetic diversity in two groups of cucumber. **a** TreeMix analysis of the inferred relationships among seven cucumber groups. Migration edges are colored according to percent ancestry received from the donor population. Scale bar shows 10 times the average standard error of the estimated entries in the sample covariance matrix. **b** LD decay of seven groups of cucumber measured by *r*^2^ implemented in PopLDdecay software^55^. **c** Nucleotide diversity of seven groups of cucumber measured by the *π* value, taking into account a 50 kb window and 5 kb sliding steps. **d** Population divergence (*F*_ST_) in the 56 artificial inbred lines

Using the TreeMix^32^ model and the seven populations, we detected that G1 and G2 were phylogenetically similar to the EA population, and the migration edge signaled introgression of EU into G1 and G2, especially to G2 (Fig. 2a), when admixture was modeled to include two migrations, suggesting that EU cucumbers were the donors used to improve G1 and G2 by breeding. This result perfectly matched our selection schemes (Supplementary Table 1). When admixture was modeled to include five migrations, we detected that migration edges to EA, G1, and G2 lines were from XSBN. Surprisingly, we did not find any introgression from INW lines (Supplementary Fig. 4). These signals of genetic introgression were supported by D-statistic analyses (ABBA-BABA tests)^33^ (Supplementary Table 2). These results suggested that G1 and G2 have experienced gene flow from other cucumber populations, which coincided with our breeding history in which G1 was improved based on the EA background and G2 was selected to change fruit traits mainly by crossing with the EU group. We also examined the residuals of the model fit to identify the relationships that were not captured by the maximum likelihood trees; the XSBN and EA groups stood out (Supplementary Fig. 5).

Increased linkage disequilibrium (LD) and reduced nucleotide diversity are expected in artificial selection lines^34^. To understand the specific LD block patterns in our 56 bred cucumbers, the LD decay (*r*^2^) with increasing physical distance between SNPs was calculated by combining the 115 publicly available lines. The results indicated that both G1 and G2 cucumbers exhibited higher LD than previously reported for the 115 lines (Fig. 2b), suggesting that large introgressions were selected and fixed in G1 and G2. We then evaluated nucleotide diversity at the whole-genome level within different groups. The average values of genome-wide nucleotide diversity (*π*) for the G1, G2, EA, EU, IND, INW, and XSBN lines were 1.56 × 10^−3^, 1.71 × 10^−3^, 1.37 × 10^−3^, 2.26 × 10^−3^, 4.25 × 10^−3^, 9.27 × 10^−3^, and 1.37 × 10^−3^, respectively (Fig. 2c). The *π* values of the INW and IND groups were higher than those of the other groups, consistent with previously published data^22^, while those of both G1 and G2 were significantly lower (*t*-test, *P* *<* 2 × 10^−16^). The high LD and low *π* values confirmed that G1 and G2 are highly artificially selected populations.

Resequencing the 56 artificially selected cucumber lines also provided opportunities to investigate selection footprints reflecting the complex genomic and genetic variations resulting from cucumber breeding. LD and nucleotide diversity analysis demonstrated that G1 and G2 were exposed to higher selection pressure, while CLR analysis revealed clear differentiation via selection between the two groups and multiple targets of selection in G1 and G2. In addition, we found that the EU group was most likely the founder cultivar for G1 and G2, especially for G2. In addition, it is worth mentioning that migration edges to EA, G1, and G2 lines were from XSBN (Supplementary Fig. 4). This can be explained by the fact that XSBN is a semiwild landrace with some unique traits that are very useful for cucumber breeding^35,36^. However, wild species of *Cucumis* are important as potential genetic sources for breeding^37^; INW is the origin population of cucumber and thus harbors higher genomic diversity than XSBN (Fig. 2c) and can be used for breeding improvement in the future.

### Selection regions in the two groups of cucumber

**Fig. 3:**
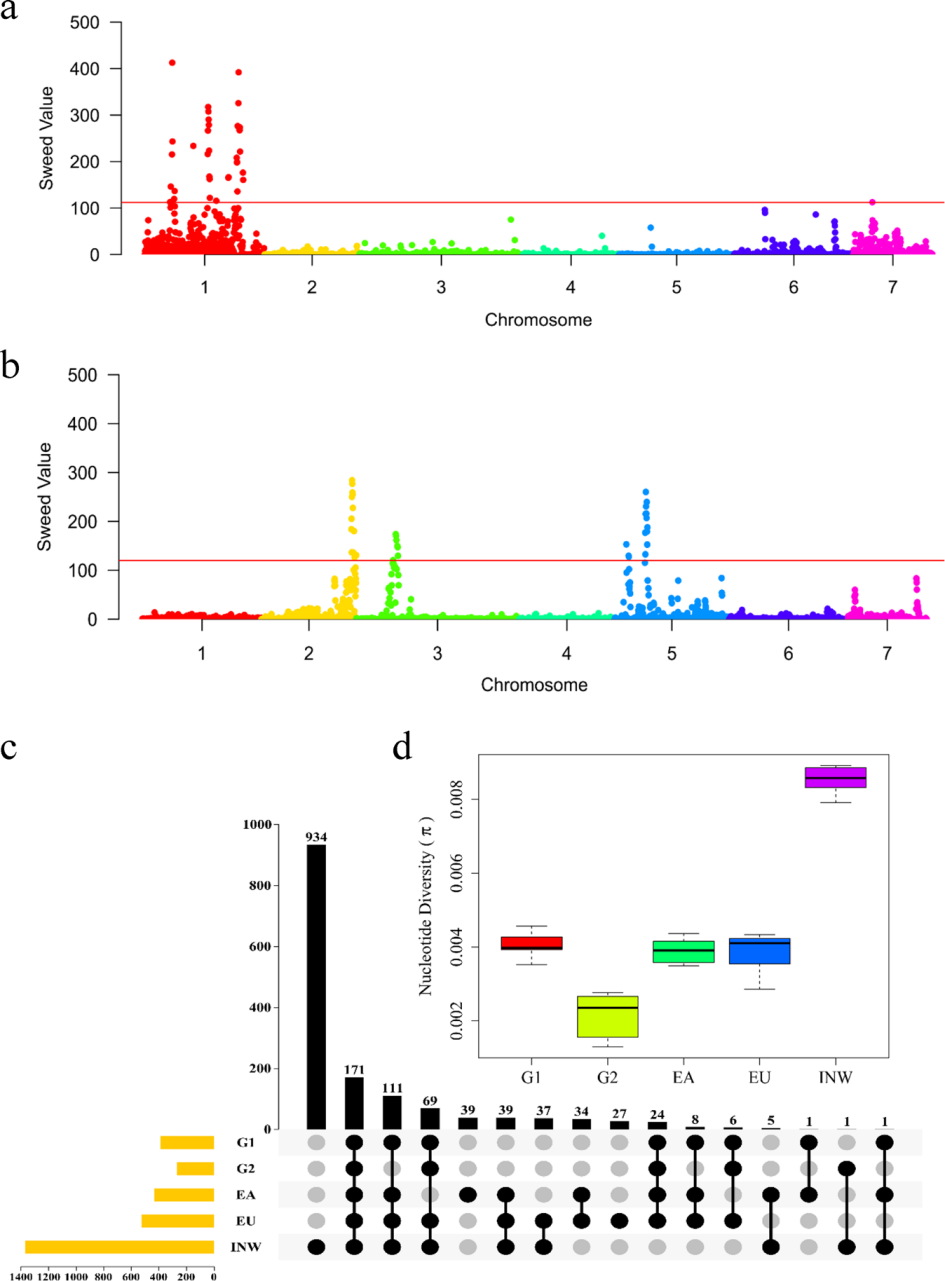
Genes under artificial selection. **a** Selection signal in cucumber group 1. Red line indicates the top 1%. **b** Selection signal in cucumber group 2. Red line indicates the top 1%. **c** Venn diagram depicting unique and shared SNPs between the G1, G2, EA, EU, and INW populations in the powdery mildew resistance QTL interval Chr1:6815000–6858200. **d** Nucleotide diversity of 5 cucumber groups in the powdery mildew resistance QTL interval Chr1:6815000–6858200, as measured by the *π* value

To assess the extent of genetic differentiation in G1 and G2, we used a method to calculate pairwise differences in allele frequency (*F*_ST_) between G1 and G2, as well as the composite likelihood ratio (CLR)^38^ to identify genomic regions differentially selected within each group. The *F*_ST_ value highlighted 89 signature sites that were distributed on chromosomes 1–6 and were significantly selected between G1 and G2 (*q* < 0.05; Fig. 2d; Supplementary Table 3). However, it is intriguing that the CLR statistics indicated selection signals over the threshold (top 1%) mainly on chromosome 1 in G1 (Fig. 3a; Supplementary Table 3) and on chromosomes 2, 3, and 5 in G2 (Fig. 3b; Supplementary Table 3). These results suggested that different target regions were under selection between the two groups during the breeding process and were consistent with different selection pressures leading to distinct groups. The selected regions of G1 and G2 detected by merging *F*_ST_ and CLR statistics contained 4074 and 3508 genes, respectively (Supplementary Table 4). Gene Ontology (GO) analysis of these genes showed enrichment in binding processes, metabolic processes and catalytic activity (Supplementary Table 5).

Previous studies identified several domestication-related genes or QTLs in cucumber, including those for disease resistance^39,40^, flowering time^41^, fruit length^42^, sex determination^42^, fruit bitterness^43^, and trichome development^28^. These reported genes or QTLs overlapped with the putative selective sweep regions detected in our study, indicating that a subset of domestication loci might have undergone selection for continuous improvement of important agronomic traits. For example, in the G1 population, we selected a soft trichome phenotype (Fig. 1 a8-S). As a result, *Csa1G056960*, which is involved in soft spine formation, was detected in the putative selected region on chromosome 1^28^ (Supplementary Table 4). Another selected trait in G1 was flowering time; *Csa1G651710*, which is responsible for flowering^41^, was also detected in the putative selected region on chromosome 1 (Supplementary Table 4). In G2, a bitterness trait was strongly selected, and two transcription factors, *Bl* and *Bt*, on chromosome 5 that regulate the pathway of biosynthesis of cucurbitacin C were detected in the selection region (Supplementary Table 4). It is known that selection imposed on the *Bt* gene during domestication led to the derivation of nonbitter cucurbits from their bitter ancestors^43^; therefore, our results proved that the target region for selection can be explained by the selection schemes applied.

The different target selection regions in G1 and G2 might reflect breeders’ preferences. In other words, most artificial selection results from the intentional breeding of target traits. However, selective breeding can also be unintentional^44^. For example, in recent decades, we did not perform a disease resistance test to screen for resistant cucumber plants, but we unintentionally kept the healthy plants and removed disease-infected plants. To date, most plants in the G1 and G2 populations have performed well in the field and have not shown any disease infection. In this study, we surprisingly found that the powdery mildew resistance major-effect QTL *Pm1.1*^39^ was located in a selection region. Then, we compared the SNPs in the *Pm1.1* interval among the G1, G2, EA, EU, and INW groups (Fig. 3c) and found that the INW, EA, and EU groups had 934, 39, and 27 unique SNPs, respectively, while there were no unique SNPs in G1 or G2. In other words, the SNPs that existed in G1 and G2 were perhaps derived from the INW, EA and EU groups and fixed by artificial selection. Interestingly, G2 had a smaller number of SNPs as well as a lower nucleotide diversity (*π* *=* 2.14 × 10^−3^, *t*-test *P* < 0.01) than the EA, EU, and INW groups (Fig. 3c, d), indicating that this interval was homozygous as a result of intensive selection and could be used to identify the causal region for powdery mildew resistance.

### Conclusions

In this study, by using next-generation resequencing technology, we identified highly informative SNPs, which were used to study the genetic relations between cucumber germplasm accessions. Here, we reported 56 artificially selected cucumber inbred lines that exhibited various breeding preference phenotypes and could be divided into two groups. In addition, the two groups exhibited distinctive genetic structure due to their breeding history involving hybridization and selection, and they were significantly different from 115 previously reported cucumber lines. Phylogenetic inference with the TreeMix model revealed a breeding history in which G1 was improved based on the EA background and G2 was selected to change fruit traits mainly by crosses with the EU group. D-statistic values were consistent with positive selection in the cucumber populations, possibly related to domestication or selection on traits of interest. We also reported the possibility that selective breeding can be unintentional. Our results revealed the molecular basis of selection during cucumber development, and the 56 selected inbred lines could be good germplasm resources for cucumber breeding programs. For example, based on specific traits, each of the inbred lines could be used to develop populations, and the highly informative SNPs are of great relevance to cucumber breeders.

## Materials and methods

### Sample collection and sequencing

The cucumber population of 56 artificially selected inbred lines was collected, crossed and inbred at the Ren Laboratory of Vegetable Science, China Agricultural University. The population was grown in an experimental field in the Changping District, Beijing. Phenotyping was conducted in Changping (N 40°13′, E 116°12′).

Genomic DNA was extracted as previously described^15^. At least 5 μg of genomic DNA was prepared from a single plant of each accession for sequencing, and a library was constructed with an insert size of ~300–350 bp for all of the lines, following the manufacturer’s instructions (Illumina HiSeq 2500, USA).

### Variant calling, filtering, and annotation

The paired-end reads were aligned against the 9930 cucumber reference genome using the MEM algorithm implemented in BWA software^45,46^ after trimming low-quality reads by using Trimmomatic^47^. Picard tools (https://broadinstitute.github.io/picard/) was employed for the removal of PCR duplicates and realignment of indel regions. Generated files in Binary Alignment Map (BAM) format were used to call single nucleotide polymorphisms (SNPs) via the HaplotypeCaller function of Genome Analysis Toolkit (GATK, version 3.8) with default parameters^48^.

To obtain high-quality SNPs, a hard filtering step was performed by following the GATK Best Practices recommendations^48^. The genotype calls were then improved by imputing and phasing the polymorphism dataset with Beagle 4.1 software based on genotype likelihoods^49^. Finally, the effects of SNPs were predicted with SnpEff 4.3 software^50^.

### Investigating population genetic structure

We further thinned the SNP dataset by using the following rules: (1) core chromosomes (“--chr 1–7”); (2) minor allele frequency > 0.05; (3) missing values <10%; (4) a Hardy-Weinberg equilibrium test (“--hwe 0.001”); and (5) pruning SNPs by “--indep-pairwise 50 10 0.1”. Two parameters (“--pca” and “--distance-matrix”) in PLINK software^51^ were used to calculate pairwise matrixes for the construction of a principal component analysis (PCA) plot and neighbor-joining tree, respectively. In addition, the ADMIXTURE (version 1.3.0) tool^52,53^ was employed to estimate individual ancestry by setting the *K*-value from 2 to 7. Nucleotide diversity measured by the π value for each population was estimated by using VCFtools^54^ with a 50 kb window and 5 kb sliding steps. In addition, the decay of linkage disequilibrium (LD) with physical distance between SNPs was calculated and visualized by using PopLDdecay software^55^. Furthermore, TreeMix-1.12^32^ was used to model the genetic drift of genome-wide allele frequency data to infer population splitting and mixing. The standard errors of migration proportions were calculated by using the “-se” option. Migration edges (with the “-m” option) were added gradually from 0 to 5.

### Identifying selective sweeps

The difference in allele frequency between the G1 and G2 groups was estimated with BayeScan software (version 2.0)^56^, which decomposed locus-population *F*_ST_ coefficients into a population-specific component (*β* value) and a locus-specific component (*α* value) using logistic regression. A positive *α* value suggests diversifying selection, whereas a negative value indicates balancing or purifying selection. A *q*-value <0.05, analogous to the *p* value but used under multiple testing, was used to indicate significance. BayeScan software^56^ was run under the default parameters. To detect regions under complete selection within the population, the SweeD program was employed to calculate the composite likelihood ratio (CLR)^38,57^, which identifies regions with significant deviations from the neutral site frequency spectrum (SFS). We set the top 1% as a significance threshold. Finally, the overlapping regions detected by *F*_ST_ and the CLR were identified as having signatures of selection. Gene Ontology (GO) annotations were assigned using AgriGO for cucumber (http://systemsbiology.cau.edu.cn).

## Supplementary information

Suplementary figures

The list of cucumber lines sampled in this study

D statistic and numbers of ABBA, BABA sites

Regions under selection in the 56 inbred lines

Genes under selection in the 56 inbred lines

GO analisis for selected genes
