# Supplementary material for: Selection footprints reflect genomic changes associated with breeding efforts in 56 cucumber inbred lines
Source: Hortic Res. 2019 Nov 15;6:127. doi: 10.1038/s41438-019-0209-4 (PMC6856066; doi:10.1038/s41438-019-0209-4)
Supplement: Supplementary file 1 — Suplementary figures [file 41438_2019_209_MOESM1_ESM.pdf]

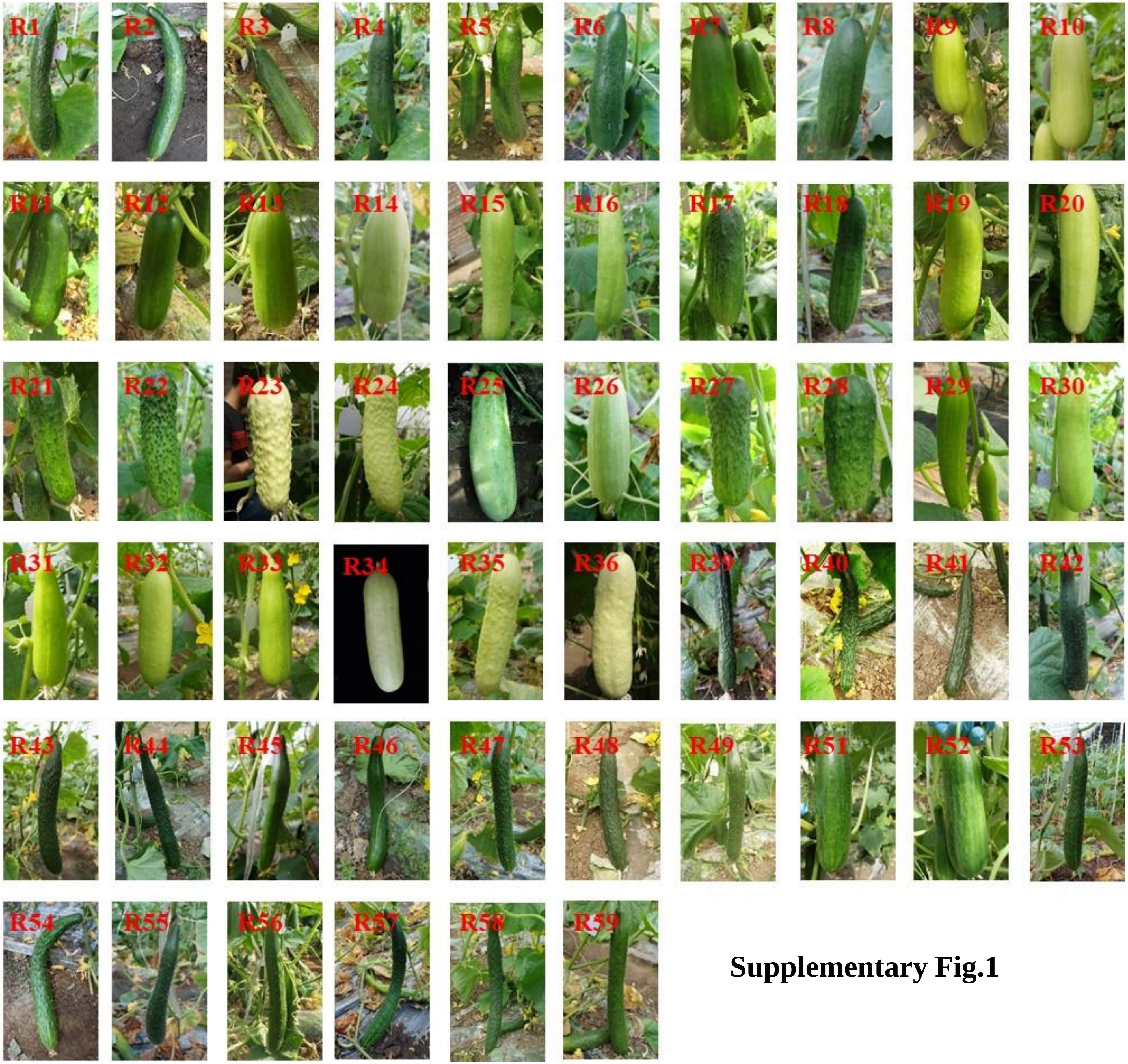

Supplementary Fig.1

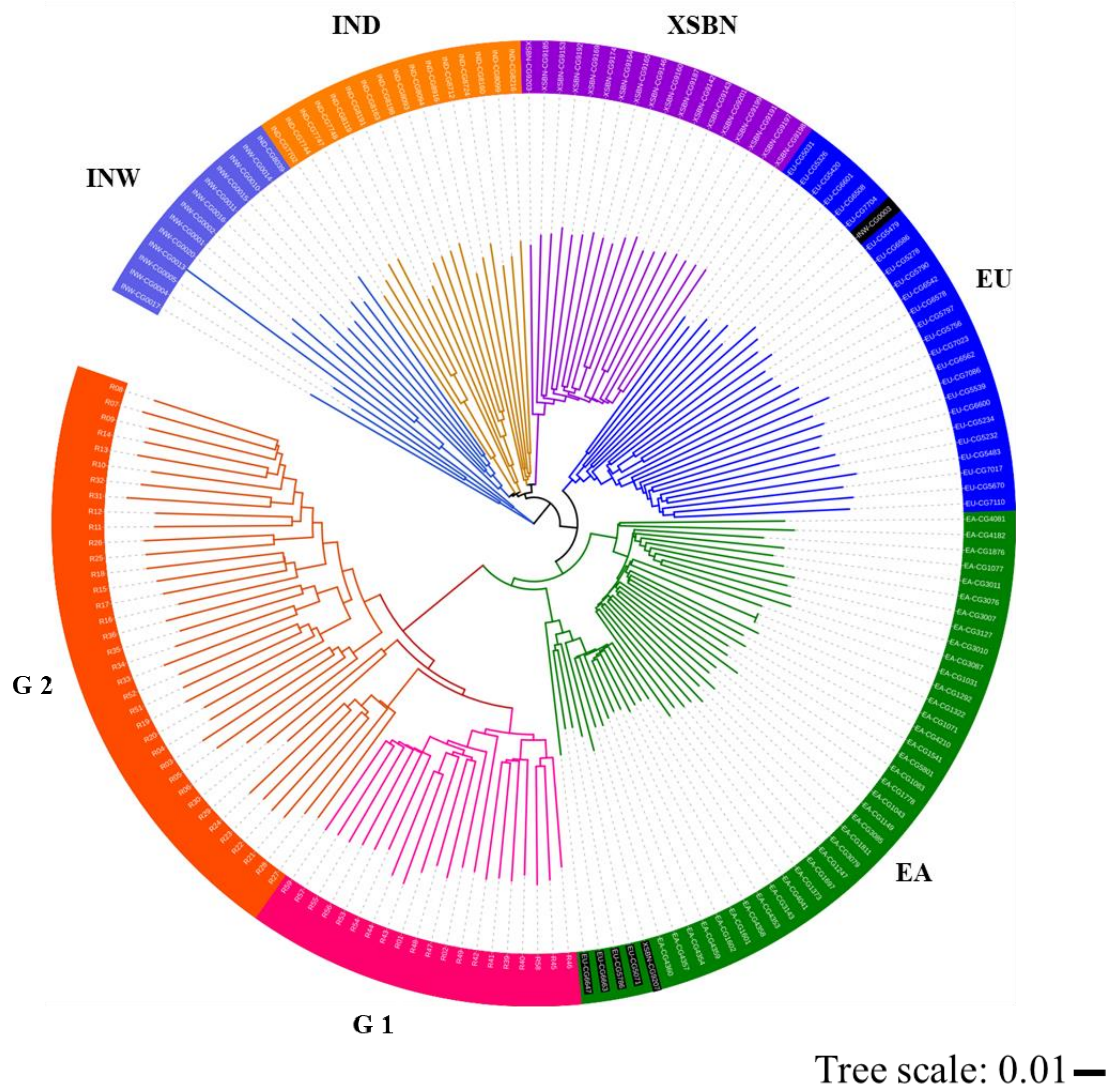

Supplementary Fig.2

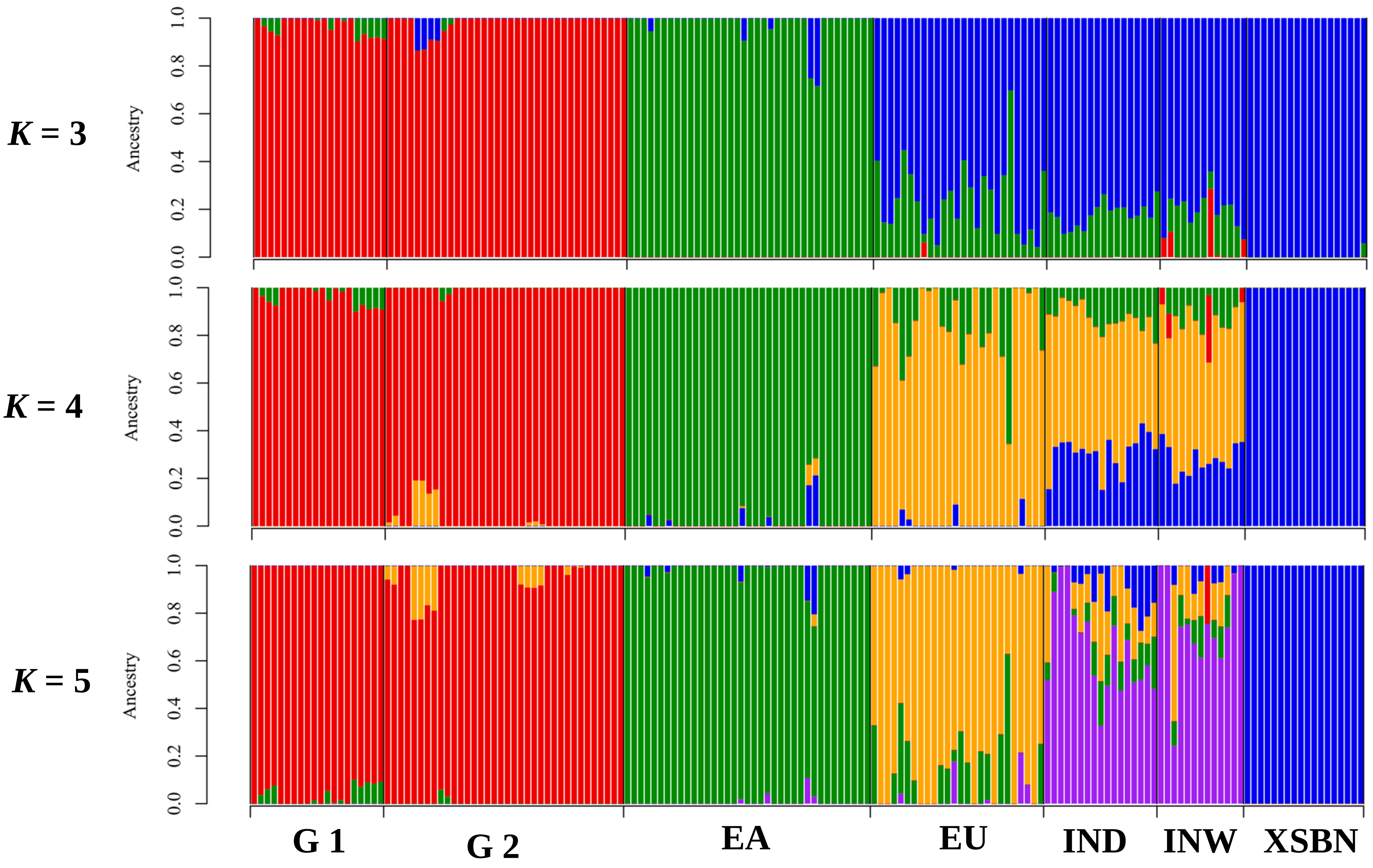

Supplementary Fig.3

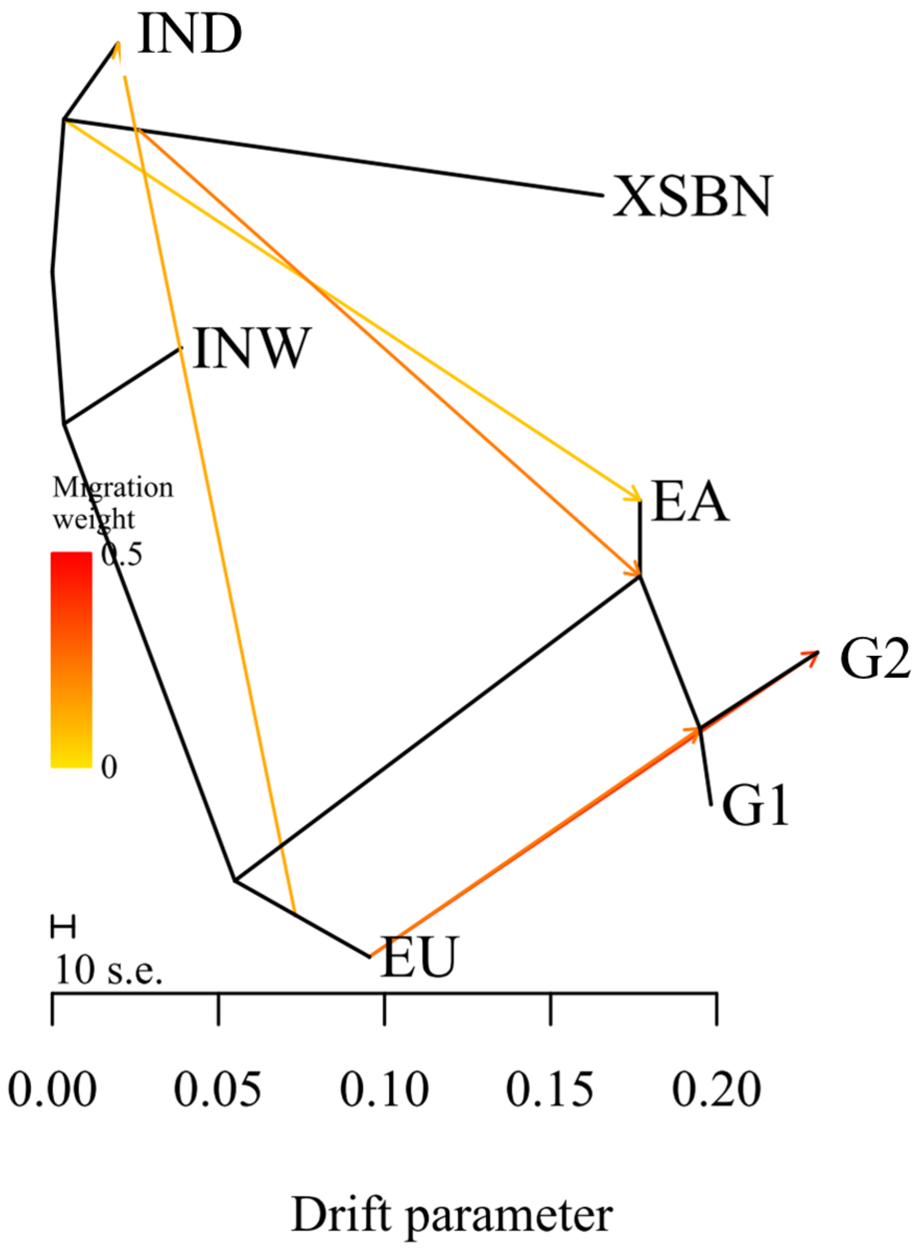

Supplementary Fig.4

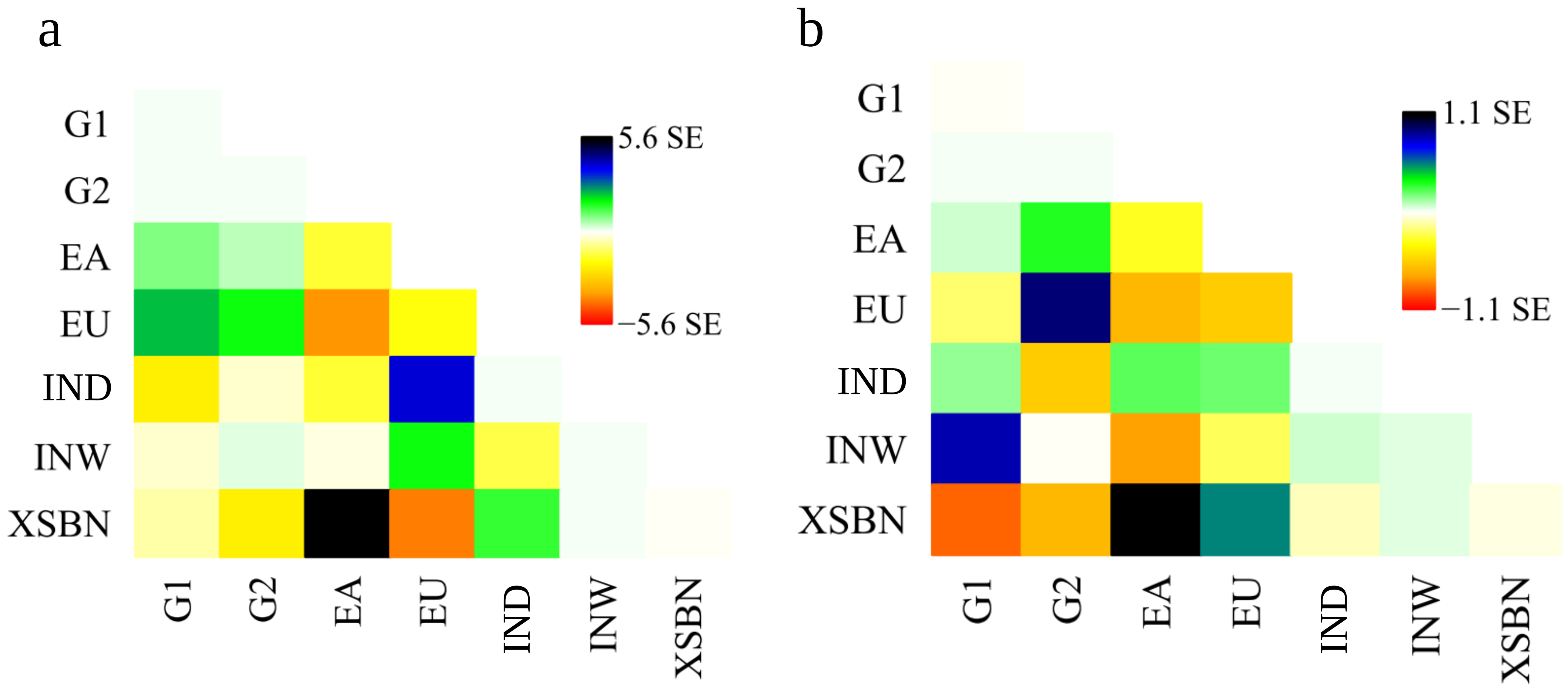

Supplementary Fig.5
